# An Osmotic Model of the Growing Pollen Tube

**DOI:** 10.1371/journal.pone.0036585

**Published:** 2012-05-16

**Authors:** Adrian E. Hill, Bruria Shachar-Hill, Jeremy N. Skepper, Janet Powell, Yair Shachar-Hill

**Affiliations:** 1 Department of Physiology, Development and Neuroscience, Cambridge University, Cambridge, United Kingdom; 2 Multi-Imaging Centre, Cambridge University, Cambridge, United Kingdom; 3 Department of Plant Biology, Michigan State University, East Lansing, Michigan, United States of America; UMass, United States of America

## Abstract

Pollen tube growth is central to the sexual reproduction of plants and is a longstanding model for cellular tip growth. For rapid tip growth, cell wall deposition and hardening must balance the rate of osmotic water uptake, and this involves the control of turgor pressure. Pressure contributes directly to both the driving force for water entry and tip expansion causing thinning of wall material. Understanding tip growth requires an analysis of the coordination of these processes and their regulation. Here we develop a quantitative physiological model which includes water entry by osmosis, the incorporation of cell wall material and the spreading of that material as a film at the tip. Parameters of the model have been determined from the literature and from measurements, by light, confocal and electron microscopy, together with results from experiments made on dye entry and plasmolysis in *Lilium longiflorum*. The model yields values of variables such as osmotic and turgor pressure, growth rates and wall thickness. The model and its predictive capacity were tested by comparing programmed simulations with experimental observations following perturbations of the growth medium. The model explains the role of turgor pressure and its observed constancy during oscillations; the stability of wall thickness under different conditions, without which the cell would burst; and some surprising properties such as the need for restricting osmotic permeability to a constant area near the tip, which was experimentally confirmed. To achieve both constancy of pressure and wall thickness under the range of conditions observed in steady-state growth the model reveals the need for a sensor that detects the driving potential for water entry and controls the deposition rate of wall material at the tip.

## Introduction

Tip growth is a specialized form of cell growth shown by higher plant pollen cells and root hairs [1], [2], fungi [3], ferns [4], bryophytes and certain algae [5] and bacteria [6]. The pollen of higher plants can show extremely high growth rates (up to 25 µm min^−1^) [7], [8] and are involved exclusively in fertilization as opposed to water and nutrient acquisition for the parent body. In view of the importance of this specialized growth pattern, a large body of data has been collected over the last few decades which illustrates the complexity of tip growth. What has emerged is a view of tip elongation, as opposed to cell expansion growth, which poses several regulatory challenges. The very fast growth rate requires a concerted delivery of material to the tip [9], [10]; the wall, which shows small localized variations in thickness during growth only at the extreme apex [11], [12] has to be stabilized against over thickening or thinning to bursting [13]–[15]; and water and osmotic solutes have to be accumulated at high rates. Furthermore, when growing in vivo the cell has to be steered in response to extracellular signals [16]–[21] i.e. pollen growth is tropic and the high growth rate has probably evolved in response to competition for fertilization between gametes. An analysis of pollen tube growth is therefore central to understanding both fertilization in angiosperms and the main elements of tip growth in general.

Different aspects of tip growth in pollen tubes have been addressed experimentally by a range of microscopic, molecular genetic and biophysical techniques (for reviews see [1], [2], [5], [9], [17], [22]–[37]) and relevant parts of these studies will be discussed in various contexts below. Quantitative models are needed to test hypotheses about tip growth and the ones published to date are of several types. (i) The morphology of the growing cell and the generation of tip shape by pressure. These models are sometimes applied to general plant cell growth (the ‘morphodynamics’ of development) [38], [39], but also specifically to tip growth mechanics, involving the expansion of the tip wall by pressure to give the shape and growth patterns observed by quantitative microscopy [3], [12], [31], [40]–[46]. (ii) The cytology of the cell tip region and the supply of wall material, the ‘vesicle supply model’ [3], [47]–[49]. (iii) The ionic relations of the cell in terms of the known pumps and transporters and their possible role in current flow and oscillations. Recent modelling of possible and probable ion transporters in the tip and shank of the pollen cell regarded as distinct areas of import and export does not indicate whether net ion accumulation is the basis of osmotic pressure in the cell, although it may contribute under certain conditions [50]. (iv) Oscillatory growth rates, both generally in plant cells [51] and specifically in pollen tubes [52].

These studies are based on a set of assumptions with parameters taken from experiments and use computation to test their fit to the experimental behaviour and thus the validity of the assumptions. They originate from an interest in different aspects of the growth process. There are as yet none which deal with the osmotic relations of the whole pollen cell, deriving the osmotic and turgor pressures as the basic driving forces and coupling volume entry with linear growth and tip expansion; such a model forms the basis of the current paper and contributes answers to problems that have not been answered by previous work.

An important aspect of pollen growth that requires explanation is the control of the wall thickness and its apparent constancy. The majority of plant growth models have to do with extension growth which was treated in a classic paper of Lockhart [53]; the model described there is based on the following two assumptions: the cell behaves as a simple osmometer and the cell wall behaves as a linear viscoelastic polymer, whose thickness is maintained constant by the continuous deposition of new materials. However, the growth rate is not constant and increases exponentially i.e. in proportion to cell length, although different patterns can be obtained by modifying parameters such as the maintenance of osmotic pressure [53] and making extended assumptions about the physics of the wall material which can change with time and turgor pressure [54].

These two assumptions apply to the model described here, but growth rates of pollen cells are steady and can be virtually constant over large increases (100–300%) in length. It will be clear that the extension model, in which cell shape and differentiation are not considered, does not apply to tip growth in several ways: (i) Growth is confined to the apex and does not involve any further linear extension of wall material already laid down behind the tip, though wall components are added to it; there is no loosening of this wall to permit linear growth by intercalation. (ii) The extreme tip is considered to be a viscous liquid polymer being stretched in a steady-state, and which shows no non-linear yield under pressure. (iii) The osmotic and turgor pressures may determine the rate of wall incorporation at the tip. (iv) The cell membrane area available for osmotic water entry has to be constant for constant linear growth. These differences, which are applied within a cylindrical geometry led by a constantly renewed tip are sufficient to require the different treatment outlined below.

There is evidence that primary wall, secreted by vesicles, behaves as a viscoplastic material that is being expanded at a high rate by the pressure in the cell and, at the same time, being continuously hardened [43], [55]. The viscous liquid film at the tip is therefore in a steady-state operating on a knife-edge between over-thickening and bringing growth to a halt or bursting when the film thins below a critical value. How this stability is achieved has been unclear; in experiments the tip wall can be caused to thicken or the cell to burst under extreme conditions but during normal growth and with moderate changes the thickness is apparently maintained within a narrow range. A further related problem is that the entry of water by osmosis has to be matched to the production of new tip wall. Here again a mismatch would lead to bursting or excess wall thickening and a cessation of growth. These considerations require that the cell’s physico-chemical relations are either intrinsically self regulating or controlled by a feedback regulatory system.

Another central but unresolved problem is the role of turgor pressure in extension growth. There is no simple relationship between turgor and growth, as highlighted by the demonstration that in some fungal cells showing tip growth there is no detectable turgor after hyperosmotic stress but growth rates are quite appreciable [56] and the observation that in pollen tubes there is no measurable turgor oscillation during oscillatory growth [14], [21]. In a characean cell study with a turgor clamp the rate of growth was shown not to depend on turgor, but turgor was necessary for growth above a threshold [57]. As turgor is the only force that can mechanically expand the new wall material, apart from the suggestion that tube growth is an amoeboid-like movement propelled by cell motors [58], [59], this question has arisen repeatedly in the literature.

Here we present a model that aims to answer some of the central questions about growth rates, wall incorporation and water uptake by considering them together as an integrated set of equations representing a single growing pollen cell. In conjunction with experimental measurements, a computer program of the model yields some clear and basic results. Given values for its key parameters derived from the literature and our own measurements, this model accurately portrays steady state growth. This state can then be perturbed by changing the values of external parameters such as external osmotic pressure and the predicted effects are compared with experimental results. The basic elements are: (i) a delivery of primary wall material; (ii) its expansion by turgor, a process which feeds area to the tip; (iii) a hardening mechanism within a tip region; (iv) an entry of water (volume), under the thermodynamic driving forces, *via* an osmotic conductance of the membrane, and (v) the presence or absence of a sensor/regulator mechanism that senses one of several potential inputs and controls the rate of wall material delivery or other potential outputs.

Our results provide a quantitative mechanistic explanation of the role of turgor pressure: how it is lower than the osmotic pressure (usually by 50–70%) and which can be very low indeed during fast growth, and why it is not seen to vary during oscillations in growth rate. The model also explains the constancy of cell wall thickness under different conditions, the growth and pressure responses to perturbations of external osmotic strength and cell wall hardening rate, and correctly predicts that water permeability and entry is dominated (even for long tubes) by the tip region. The model also strongly suggests that a sensor reacting to water potential is required to stabilize pressure and growth by regulating wall deposition.

## Results

### Model Assumptions

**Figure 1:**
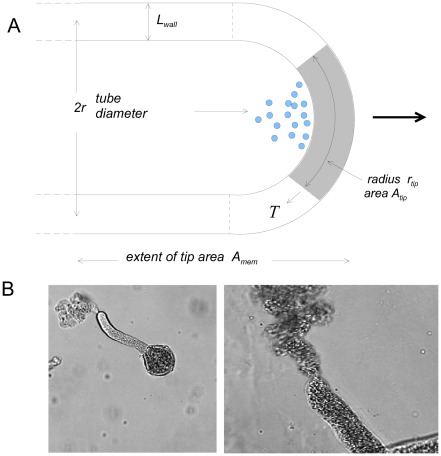
Diagram of the pollen tube tip and the rupture of the tip disc. **A**: Diagram of the tip showing the zone of cell wall exocytosis by vesicles (in blue) and the pectin disc where new wall material is stretched by tension (*T*); also labelled are the tube and tip radii (*r* and *r_tip_* respectively), tip zone area (*A_tip_*), wall thickness (*L_wall_*), and surface area of the osmotically active zone (*A_mem_*). **B**: Pollen tubes of *Mahonia* (left) and *Lilium* (right) showing the small tip area that bursts under hypotonic shock or calcium removal.

**Figure 2:**
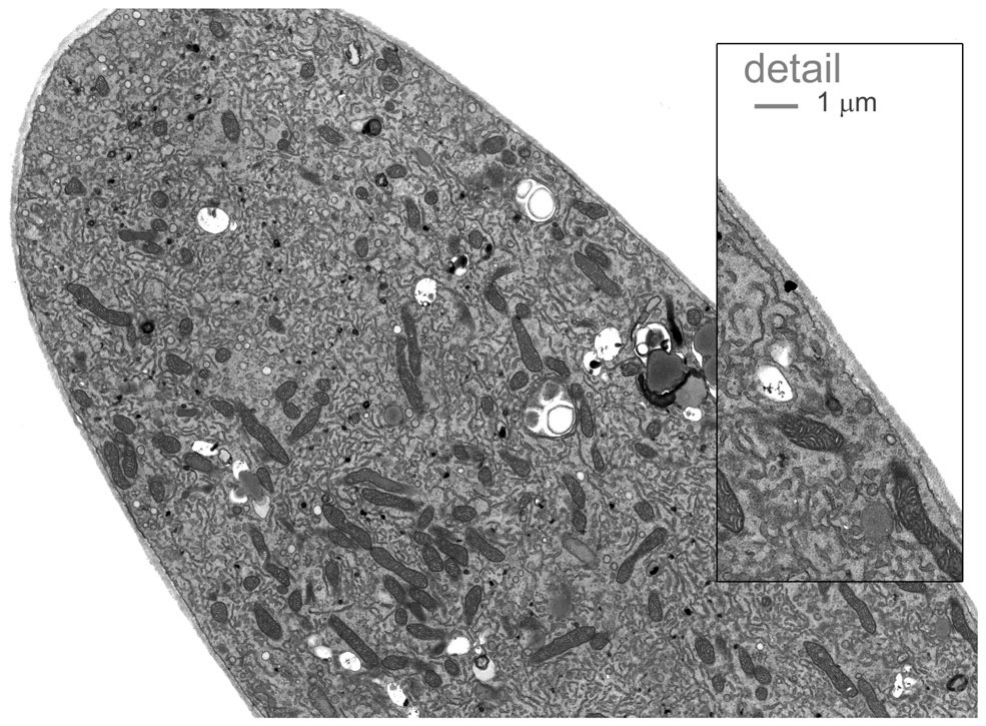
Transmission electron micrograph (TEM) of the tip of a growing *Lillum* pollen tube. (TEM, bar = 5 µm) with enlargement of part of the image *in situ* showing the tube wall. Pollen was germinated on solidified medium for 3–4 hrs and then fixed and prepared for TEM as described in the Methods section.

Extrusion of material at the tip is by vesicular delivery of highly methylated pectin as a viscous aqueous solution which is then demethylated [9], [36], [60] and cross-linked by extracellular Calcium ions [61]. We treat this secreted solution as a liquid film whose initial viscosity is rapidly increased by cross-linkage. Tip growth is considered as the expansion of a film with hardening, not a loosening of cell wall material possessing an elastic modulus, as is the case for extension growth in plant cells. The viscoelastic [43] or viscoplastic [55] properties of the film are unclear, but can be dealt with in a simple way by introducing a form of tension *γ*, not necessarily interfacial. The model here relies on an isotropic treatment of a pectin tip disc which is not yet strengthened by other wall material [62], [63] and which captures the essentials of the extrusion-expansion process in its early stage, determining the growth rate and stability of the tip. Its shape and anisotropy, controlled by spatial biochemical modifications [41], is taken as given. A more detailed viscoplastic approach to modelling tip shape is compatible with an isotropic treatment [43]. Here pectin exocytosis is confined to a circular axial disc (Figure 1A) with radius of curvature *r_c_* and disc radius *r_tip_*. Its area *A_tip_* collects all the extruded pectin and generates wall area growth and thereby the linear tube growth rate. For simplicity, we take the curvature *r_c_* to equal the cross-sectional radius *r* of the pollen tube, *r_c_* = *r* = 8 µm, with a hemispherical tip of disc radius *r_tip_* = 2 µm, the extrusion zone being a small cap at the apex. The disc area *A_tip_* does not need to be known exactly as it can be varied without perturbing either the rate of area expansion at the tip, growth rate or other parameters providing that the extrusion rate of pectin into the disc remains in proportion to *A_tip_*. We have used an area estimated from tip bursting experiments (below) which is large enough to encompass the pectin extrusion. This is equivalent to assuming that the disc is well-mixed with respect to native and cross-linking material and also that the wall is not thinning outside the disc, but being distributed over subsequent annular cross-sections of the tip at a diminishing axial rate until it becomes the shank wall; the geometry is then constant (Figure 2).

The situation is complicated by the interaction between film thickness and the hardening process. We here assume that the hardening is controlled by (i) the rate of carboxyl group exposure by demethylation and (ii) the cross-linking rate due to calcium ions. In stretching experiments with pectin films the viscosity can be modulated by calcium in just this way to control the rates of stretching by an applied force [61]. The hardening rate *α* has been derived from the assumption that the rate-limiting factor is the diffusion of calcium ions into the new wall governed by a diffusion-fixation equation; we have not attempted to model the demethylation pattern within the new wall but assumed that demethylated pectin is produced concomitantly with extrusion and that the binding sites for calcium are evenly distributed (Figures S1 and S2). More complex models of pectin equilibration have been suggested but with no experimental justification at present [64].

### The Model

Water enters the tube at a rate that depends on the driving forces (osmotic and hydrostatic pressures), the osmotic permeability (of the membrane plus wall) and the surface area
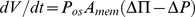
(1)where *dV/dt* is the rate of intracellular volume increase, *P_os_* is the osmotic permeability, *A_mem_* is the membrane area over which water enters or leaves the tube, and ΔΠ and Δ*P* are the positive osmotic and turgor pressure differences between the intracellular (*i*) and external phases (*o*) equal to (Π*_i_* - Π*_o_*) and (*P_i_*–*P_o_*) respectively.

The volume of the tube, and therefore its surface area and length, are related geometrically (cylinder volume is *πr^2^L* and its surface area is 2*πrL*)
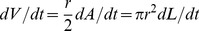
(2)where *r* is the radius of the tube, *A* is the surface area of the tube shank and *L* is the tube length.

The internal osmotic pressure Π*_i_* increases at a rate determined by the difference between osmolyte accumulation and dilution by water entry
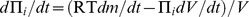
(3)which is obtained from the time derivative of *m = cV* where *m* is the osmolyte content, *c* the concentration, *dm/dt* the rate of solute generation generated by membrane transport or chemical reactions from storage pools, and Π*_i_* = *c*RT where RT has its usual meaning. The solute generation rate drives the value of Π*_i_*, determining *dV/dt* and increasing *P_i_* which provides the force for expansion of the tip. *dm/dt* is therefore a fundamental parameter that determines the growth rate of the pollen tube.

The increase in cell surface area occurs at the tip apex (Figure 1A) where the rate of pectin expansion depends on the tension *T* exerted, the balance between the surface tension of the tip wall material and the stretching force generated by the turgor pressure. This is a dynamic steady-state during growth, analogous to an expanding section of a soap bubble that is being fed with new liquid from the inside. The area over which the stretching takes place is modelled as a circular cap of radius *r_tip_* whose size is taken to be approximately that of the tip area that ruptures during bursting and whose radius is taken as *r,* the pollen tube radius; this area is substantially less than that of the whole curved tip (Figure 1B).

We require a stress-strain relationship for the stretching of a curved viscous liquid disk of radius *r_tip_* and radius of curvature *r*. The strain rate is (*dA_tip_/dt)/A_tip_*, where *A_tip_* is the area of the disk at the tip, and the stress, for a tangential force *F* acting outwards on the disc of thickness *L_wall_* and perimeter *2πr_tip_* is equal to *F/2πr_tip_ L_wall_* or *T/L_wall_* where *T* is the tension. The proportionality coefficient is 1/*η*, where *η* is the viscosity of the material. The relation is therefore (*dA_tip_/dt)/A_tip_* = *T/ηL_wall_*. The tension *T* is given by a Young-Laplace expression for the difference between the tangential tension due to the pressure *ΔPr/2* (stretching the film) and the surface tension *γ* (tending to contract the film) i.e. (*ΔPr/2 - γ*). We therefore have
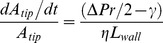
(4)The viscosity *η* of the cell wall material at the tip, which governs the stiffness, depends on the initial viscosity of the extruded material (liquid pectin suspension), the rate of hardening and the rate of addition of new material
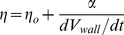
(5)where ηo is the initial viscosity and the last term is an incremental viscosity. α is a pectin hardening rate which is proportional to the bound calcium ion concentration, and dVwall/dt is the volume extrusion rate of the pectin sol into the wall; the form of Eq.5 is derived in Supporting Information S1. The expression represents the balance between extrusion and calcium hardening rates as an additive term to the initial viscosity. It can be seen that when α is high so is the viscosity, and this lowers the area expansion in Eq.4, but if the hardening is inhibited (α = 0), for example by inhibiting demethylation or removing calcium, then the native initial viscosity dominates the spreading process, which is then expected to be very fast, threatening tip stability.

The balance between the rates of tip stretching and addition of material to the cell wall determines the thickness of the tip wall by conservation of volume (i.e. the time derivative of volume as area x thickness)
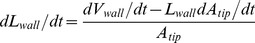
(6)where *dL_wall/_dt* is the rate of wall thickening. Finally, a sensor equation embodies the postulated relationship between what is sensed (here the water potential difference or driving force for water entry) and what is regulated (here the rate of deposition of cell wall material at the tip)
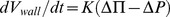
(7)where K is the coupling constant or signal amplification factor between signal input and output. The input (ΔΠ−ΔP) might seem an awkward compound variable to sense but in fact it is quite natural for a semi-permeable water pore; we do not go into the thermodynamics of this here [65], [66]. It has the immediate effect of setting a growth rate only when there is an entry of water to the cell i.e. (ΔΠ−ΔP)>0. We envisage this sensor as spanning the membrane and having access to both inner and outer phases and being impermeable to the major osmolytes. In most of the simulations of this paper Eq.7 was used as shown here. However, different sensor output relationships were tested including no sensor where dVwall/dt = constant, representing a fixed rate of deposition.

### Turgor and Osmotic Pressures

#### Turgor pressure is naturally lower than osmotic pressure

Rearranging Eq.1 yields.
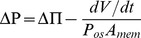
(8)


Thus an osmotic pressure driving water into the cell during growth is associated with a turgor pressure that is less than the osmotic pressure at all growth rates. Only when growth is zero (*dV/dt* = 0) does Δ*P* = ΔΠ (water potential is equal inside and outside the cell). The water also approaches equilibrium when the osmotic conductance *P_os_A_mem_* is very high.

#### Simulations

**Figure 3:**
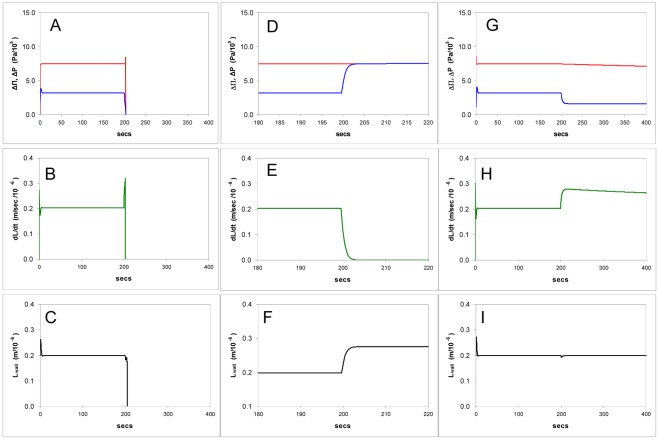
Simulations of the model showing computed time courses of osmotic and hydrostatic pressures, cell growth rate, and cell wall thickness in response to changes in the wall hardening factor *α*. **A-C**: The effect of dropping *α* to zero, the equivalent of removing external calcium, (so the wall strength has the value for pectin without cross linking): hydrostatic and osmotic pressure gradients fall to zero as the cell bursts, growth rate becomes unstable and the cell wall bursts as its thickness falls to zero. **D-F**: Hardening is increased twenty fold, the equivalent of raising the external calcium concentration from 0.1 to 2 mM: turgor pressure rises to equal the osmotic pressure gradient, growth rate falls to zero as the wall thickness rises. **H-I**: Hardening is lowered to 50% of the steady state value; turgor pressure falls abruptly, growth rate rises and tip thickness remains stable.

**Figure 4:**
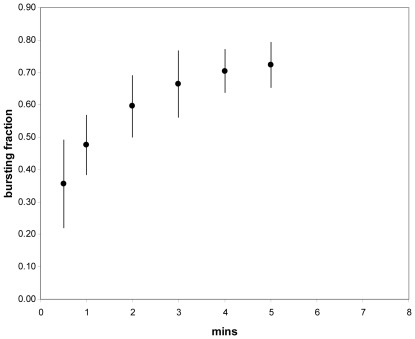
Time course of tip bursting of *Lilium* pollen tubes after removing calcium from the growth medium. Pollen was germinated and grown in basic medium, followed by perfusion with calcium-free medium containing 500 µM BAPTA. Bursting was followed by optical microscopy and the data points show the mean and standard deviation. For a total of at least 45 tubes in 3 separate experiments. *n.b.* some tubes stopped growing but did not burst; the half-time of bursting is similar to that for diffusion between the agar film containing the tubes and the flowing Ca-free medium.

**Figure 5:**
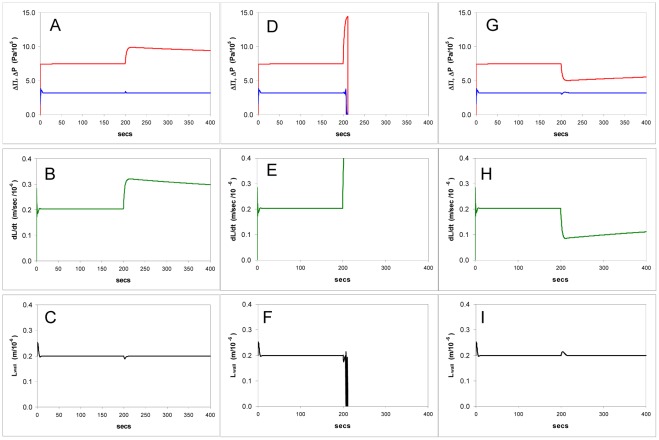
Simulations of the model showing computed time courses of the effects of changes in the osmotic pressure gradient ΔΠ (the equivalent of experimentally changing the osmotic pressure of the growth medium). **A-C**: The effect of diluting the external medium total concentration (*C_o_*) from 0.3 M to 0.2 M, showing stability of turgor pressure (A) and wall thickness(C) with an increase in growth rate (B). **D-F**: The effect of diluting *C_o_* from 0.3 M to 0.01 M (hypotonic shock), leads to bursting: a sudden increase in tip extension rate (E), wall thickness instability and bursting (F), turgor and osmotic pressure gradients first rise and then rapidly drop to zero (D). **G-I**: Decreased, by raising *C_o_* from 0.3 M to 0.4 M leading to new steady-state with lower growth but no change in pressure or wall thickness.

The last term in Eq.8 represents the discrepancy between ΔΠ and Δ*P*. The discrepancy can be seen in all simulations (e.g. Figs.3, 4 and 5). Using parameter values similar to those that we determined for *Lilium* tubes, which yield growth rates and wall thicknesses also in agreement with our observations, the turgor pressure is predicted to be about half of the osmotic pressure under steady state growth conditions: by tip plasmolysis we estimated the osmotic permeability *P_os_* to be close to 1.32×10^−3^ cm/s (9.36×10^−6^ cm/MPa.s), similar to that determined by others [67], [68], and the membrane area *A_mem_* to be 1×10^−5^ cm^2^ (discussed below in ***Osmotic water movement is confined at the tip.***); with a tube of radius 8×10^−4^ cm and growth rate of 0.2×10^−4^ cm/s the discrepancy in steady-state growth simulations was about 0.43 MPa for these values.

#### Experiments

Previous measurements of osmotic pressure made by incipient plasmolysis and of turgor pressure made with a pressure probe confirm the model’s prediction of a substantial discrepancy [14]. When the mean osmotic pressure in *Lilium* tubes was ∼0.8 MPa the turgor was ∼0.2–0.25 MPa i.e. 25–30%. The authors seek an explanation for this difference in possible inadequacies of the plasmolytic method and a similar conclusion that the plasmolytic method for determining ΔΠ must be at fault has been advanced for hyphae of water moulds [56]. However, there is no need for this in view of Eq.8. Indeed in a cell expanding by tip growth there cannot be equilibrium (ΔΠ = Δ*P*) due to the fact that a water potential difference (ΔΠ - Δ*P*) must be positive to drive water into the cell. This difference, referred to as ‘water potential’ has been pointed out in many studies to be an inescapable thermodynamic requirement for water movement into whole cells [53] and demonstrated between cells in tissues undergoing transpiration [69]–[72].

### Osmosis is Predicted to Occur in an Apical Zone

#### Simulations

**Figure 6:**
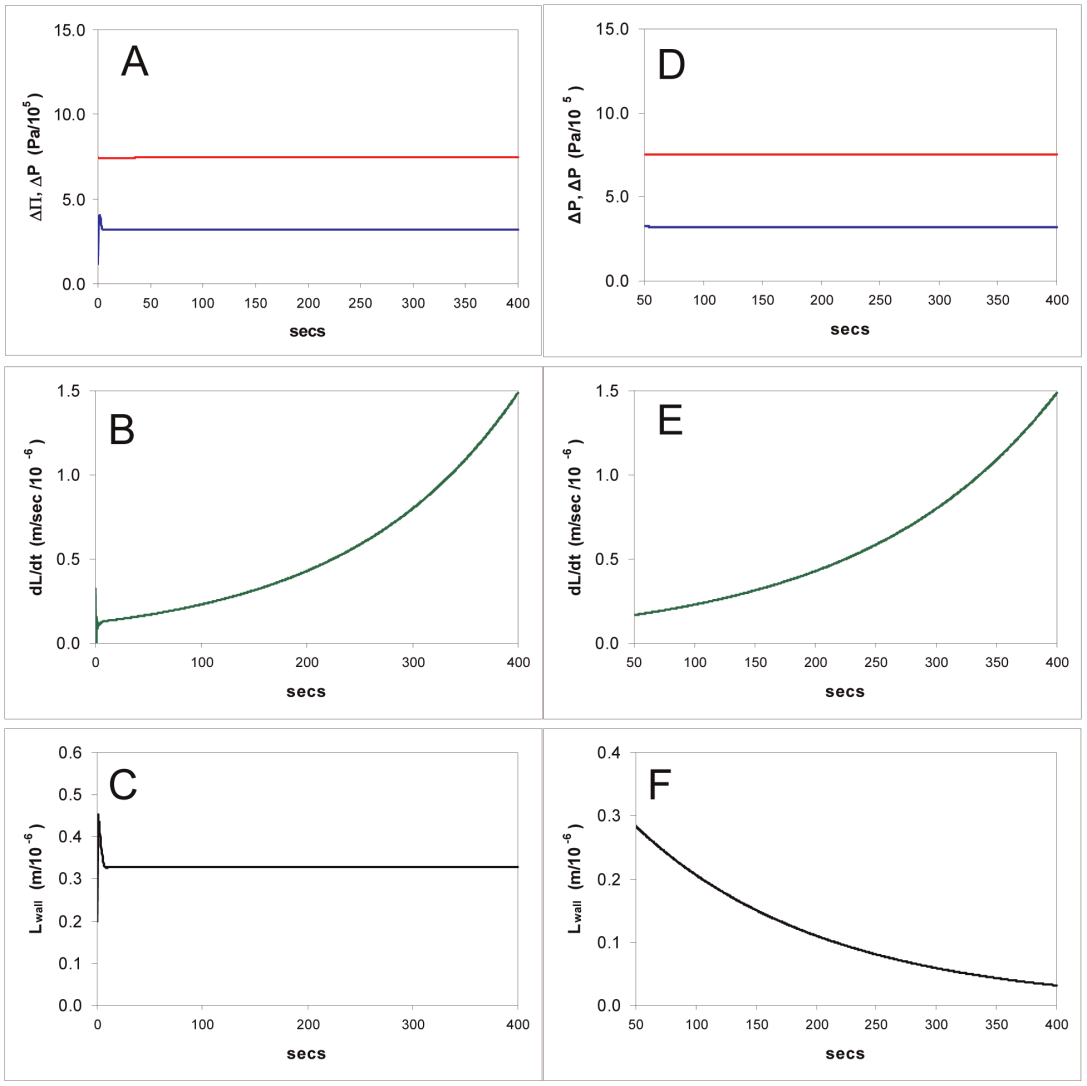
Simulation of the model showing computed time courses of the growth of cells without a constant osmotic zone length *L_perm_* and area *A_mem_*. **A-C**: Sensor feedback stabilizes tip thickness (C) but growth rate accelerates (B) indefinitely. **D-F**: No sensor feedback, the tip thins continually (F) as growth rate increases (E).

This model also highlights a hitherto neglected aspect of tip growth: Eq.1 indicates and simulations confirm that as cells grow and the area of the membrane expands so does the water permeability of the whole cell. In the simulation illustrated in Figure 6.A-C the osmotically permeable area *A_mem_* steadily grows with the area expansion of the cell (2πr.*dL/dt*) and cell membrane water permeability (*P_os_*) is assumed to be the same for the additional tube length. The turgor Δ*P* is a constant, the osmotic pressure ΔΠ rises to a plateau, and thus the driving force on the water, (ΔΠ - Δ*P*), is constant in Eq.1 - but the growth rate *dL/dt* steadily increases i.e. the cell grows faster the longer it gets. Increasing growth rates in single cells, or a positive correlation of tube length with growth rate were not observed in this study or in others [14]; the relation between growth rate (*Y*, µm/min) and tube length (*X*, µm) in our experiments was *Y* = 10^−4^ *X*+8.91, R^2^ = 10^−4^, n = 152. This behaviour is shown with the sensor controlling pectin extrusion. If this sensor is not present the same behaviour is shown but the tip now thins as the growth rate increases (Figure 6.D-F).

To allow for steady state growth, the model therefore requires that the total cellular water permeability be kept constant, or that other parameters are continuously adjusted to exactly compensate for increasing tube length. The second possibility seems unlikely because there is no evidence for ΔΠ or Δ*P* changing with tube length, so the driving force for water entry is not being adjusted; there are also no apparent changes to the wall thickness or composition (and therefore its mechanical properties). Thus to maintain steady growth rate the permeable area, *A_mem_* must also be a constant. The model therefore points to a prediction of an ‘osmotic zone’ at the tip - a membrane/wall area of defined size and permeability. The length predicted by the model, giving a similar growth rate to that observed in our experiments (∼12 µm/min) for a *Lilium* tube of 8 µm radius, is ∼20 µm.

**Figure 7:**
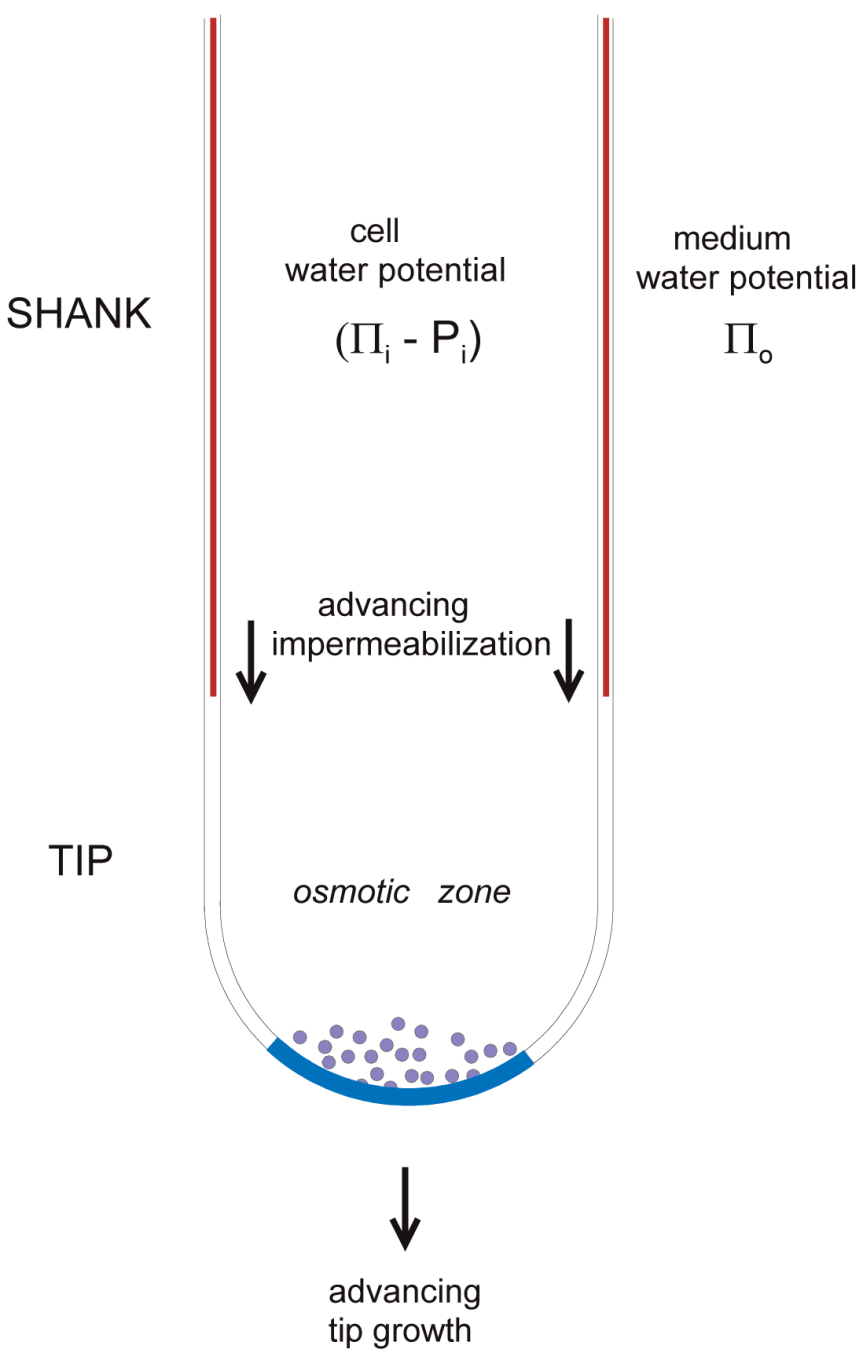
Diagram of the osmotic tip zone showing direction of growth and the following advance of impermeablization; vesicles and tip zone of pectin deposition and stretching (in blue).

There are two ways in which water permeability could be kept stable. (i) The zone is not a region of plasma membrane but is a fixed number of water pores that dominate the osmotic permeability *P_osm_*. (ii) There is a process of ‘impermeablization’ which proceeds as the tip advances. This would have to track the tip in its wake i.e. keep pace with it at a certain length behind it, thus resulting in an osmotic zone of fixed length behind the tip apex (Figure 7). We think that (i) is very unlikely because it is difficult to envisage how permeability could be constant over the lifetime of the growing cell; protein water pores do dominate *P_osm_* in plant membranes when they are present at normal densities [73], with the remainder due to permeability of lipid bilayers. If new membrane is added without water pores this would still add substantially and steadily to *P_osm_*. We present experimental evidence supporting (ii), based on dye entry and plasmolysis results.

#### Experiments: Dye entry supports a limited permeable zone

**Figure 8:**
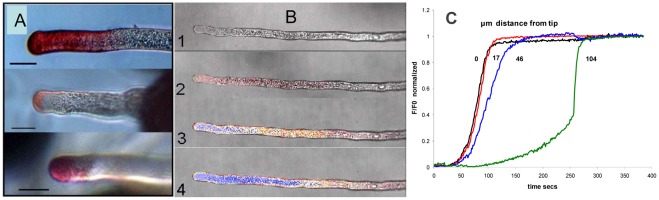
Entry of Neutral Red and Calcein AM dyes into tubes of *Lilium* growing in basic medium. **A**: Optical micrographs (scale bars 20 µm) of the front ends of growing *Lilium* pollen tubes exposed to Neutral Red dye in the medium for 60 secs followed by washout. **B**: Video micrographs showing Calcein AM entry into a growing *Lilium* tube. Images are transmission optical images from a video with the corresponding fluorescence intensity pseudo-colored (red to blue, rising concentration) images superimposed. Images B1–4 were taken at 20 sec. intervals. **C**: Time course of Calcein fluorescence intensity during entry at four distances from the tip: a - 0 µm (black); b - 17 µm (red); c - 47 µm (blue); d - 104 µm (green). A video clip of Calcein entering four tubes can be seen in Video S1.

When Neutral Red (NR) is applied to *Lilium* pollen tubes growing on agar in a flow chamber and is then washed away after 1 min it leaves the tip stained red but does not stain the rest of the tube (Figure 8A). At longer loading times, NR appears progressively down the tube from the tip region. We interpret this as entry in the apical zone followed by transfer down the tube by cytoplasmic streaming [68].

The acetoxy-methyl ester of Calcein (CAM) is lipid permeable and only fluoresces after de-esterification to calcein in the cytoplasm; the hydrolysis is fast in pollen and the calcium binding is low at cytosolic pH so its fluorescence intensity is simply a function of its own concentration. When growing cells were perfused with the dye, the time course of the rise of calcein at any distance from the tip could be monitored with video confocal microscopy (Supporting Information S1, section 4). It can be seen from measurements at four positions down a single tube that at distances from the tip less than about 20 µm the cytoplasm loads with calcein at similar rates (Figure 8.B,C). At longer distances the cell fluorescence is delayed. Together with neutral red penetration we interpret this slow spread along the tube as being due to calcein transport by cytoplasmic streaming and diffusion. The tip entry is too fast to be attributed to endocytosis.

A study of proton currents across the tip membrane [74] showed that application of gramicidin A, which forms proton channels in the membrane with immediate effect on the local trans-membrane pH, only affected pH 10–15 µm from the tip apex but not down the shank. This indicates that the shank wall is impermeable to gramicidin as well as dyes and constitutes a barrier outside the cell membrane.

#### Experiments: Osmotic water movement is largely confined at the tip

**Figure 9:**
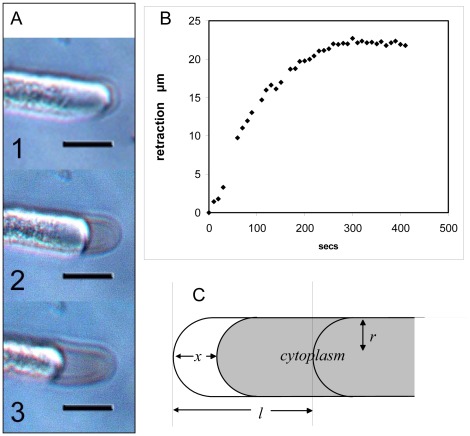
Retracting cytoplasm near the tip of a growing *Lillium* pollen tube during plasmolysis. **A**: Three video stills (1–3) at 20, 100, 300 secs (bar 20 µm). **B**: Time course of plasmolytic retraction at the tip showing the strong slowdown of retraction after the cytoplasm has retracted by approximately 20 µm. **C**: Diagram of the retraction process: the unstirred layer inside the tip wall during plasmolysis is of length *x* and the observed maximum retraction length is *l*.

If the shank is osmotically impermeable, water efflux during plasmolysis should be confined to the tip. To test this we conducted plasmolysis experiments in which growing tubes were subjected to a rapid increase in osmotic pressure of the medium (0.3 to 0.8 Osm. by sucrose addition) in a flow chamber, which induces plasmolysis. As shown in Figure 9 the cytoplasm retracted from the wall at the tip. This retraction slowed dramatically after 15–20 µm. The near cessation of retraction at the tip cannot be due to the approach to osmotic equilibration of the cell because the volume of water lost at the tip is only a very small fraction of that which would be required to bring the whole pollen cell into osmotic equilibration with the new external medium. By inspection of whole tubes at the plateau of cytoplasmic contraction we estimate shrinkage to be about 10–20% of that expected for full osmotic equilibration for a 200–500 µm tube on transition from 0.3 to 0.8 medium osmolarity. The percentage would be progressively less the longer the tube. In keeping with this, the retraction curves are independent of pollen tube length. Thus it appears that water permeability is much higher in the same tip zone where dyes enter and low along most of the tube length. Plasmolytic retraction was also seen at the pore region of the grain, but nowhere else, in keeping with the entry of neutral red at this point also.

Describing quantitatively the osmotic retraction process in terms of a high permeability zone at the tip and low permeability along the shank allows the osmotic permeability at the tip to be deduced from the retraction time course. Figure 9.C is a diagram of the process, and the form of the expected behavior is described by the equation
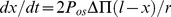
(9)(see Supporting Information S1) where *x* is the retraction, *r* the tube radius, ΔΠ the osmotic pressure difference and *P_os_* the osmotic permeability. We interpret *l* the apparent ‘retraction length’ to represent the length of the water-permeable wall at the apex constituting the osmotic zone or *L_perm_*; the rest of the shank wall is impermeable except at a small zone where it meets the pollen grain. At the start of plasmolysis the only membrane in contact with the external hyperosmotic solution is that in contact with the wall within the osmotic zone. As the cytoplasm retracts and loses contact with this wall a layer of solution forming an unstirred layer (USL) in the space between the retracting plasma membrane and the tip wall. When the membrane has shrunk away from the wall at the end of the osmotic zone further plasmolysis is greatly slowed because the shank wall from there onwards has a very low water permeability. The decline of the plasmolytic rate to a plateau is therefore explained as being due to a fall in the area of permeable wall in contact with the cytoplasm.

The USL between the apical wall and the plasma membrane is composed of stationary fluid which is not stirred by any flow or movement in the bath. In addition, the tip cell wall being a porous matrix freely permeable to small molecules and ions, the osmotic permeability is effectively zero. Further osmotic flow out of the tip is greatly reduced within the time scale of the plasmolysis, and a retraction curve of similar form to Eq.9 is obtained. To verify this analysis of plasmolytic retraction we used Eq.9 to calculate the osmotic permeability *P_os_* and compared it with values previously obtained from the *Lilium* tube cell membrane by an independent technique [67], [68].

Fitting calculated to observed shrinkage time-lapse sequences yielded values of radius *r*, retraction length *l* and permeability *P_os_* using Eq.9. The length of the osmotic zone obtained by this tip plasmolysis ranged from 15–30 µm over a range of tips with a mean close to 20 µm. The value of *P_os_* was 1.32×10^−3^±0.31 (SE) cm/s, n = 12. In a recent study of the osmotic properties of the *Lilium* tube, protoplasts were made from *Lilium* pollen tubes and used to determine *P_os_*; results ranged between 1.21–1.57×10^−3^ [68] and 1.32×10^−3^ cm/s [67] depending on the precise analysis of their swelling curves. These are free from any membrane-wall interactions and in close agreement with our value. They confirm that and strongly support the idea that pollen tube shank walls behind distal to the tip region have very low water permeabilities. If the plasmolysis rates we measured were due to water movement over the whole tube length then permeability values would have to be very low throughout as well as being dependent on total tube length, and could not explain the retraction curves such as the one shown in Figure 9.B. Relevant to this is the finding that after D_2_O has been injected into growing pollen tubes, it can be shown by Raman spectroscopy to efflux near the tip as opposed to the shank [75]. A related study of water absorption by young root hairs indicates that normally the absorbing zone is localized at the tip [76].

### The Relation Between Turgor Pressure and Growth

#### Simulations

In this model the turgor pressure has two opposing effects: (i) controlling the water entry - the primary force for growth is ultimately the osmotic pressure difference ΔΠ which drives water into the cell but the turgor pressure is outwardly directed and counteracts this, opposing the creation of new volume; (ii) controlling the area expansion of the tip wall polymers (pectin) which translates into new cell volume. In the steady state an increase in the plasticity of the wall will immediately cause the turgor pressure to drop and this effect is shown in Figure 3.H–I, which models the decrease in viscosity of the apical wall as a reduction in the hardening rate constant *α*. This has the effect of decreasing both Δ*P* and ΔΠ and slightly raising the growth rate. The turgor pressure declines to a lower level (to near zero if the wall is made progressively less viscous) whilst linear growth is modestly raised (Figure 3.H-I). This drop will increase the rate at which water enters the cell, and this exactly matches the volume increase made possible by the wall expansion, in the approach to a new steady state. This new steady state will have a lower pressure but an increased extension rate, resulting in a (potentially counterintuitive) negative relationship between the two.

#### Experiments

Eq.8 shows that the faster the water entry, and therefore the growth rate, the lower will be the hydrostatic pressure (other parameters being constant). Thus at low pressures and high growth rates ‘turgor is necessary, but not sufficient’ to explain apical extension rates [77]. Consistent with these aspects of the model are experiments in which growing *Saprolegnia* hyphae were subjected to increases in external osmotic pressure i.e. ΔΠ is decreased [56], [58] but the growth rate is maintained. This should come about by an increase in the plasticity of the wall, and this effect was demonstrated by the fact that cells burst more easily (i.e. at lower hydrostatic pressures) when injected with volume from a pressure probe after ΔΠ is decreased [78]. Eq.8 and the model simulations describe this situation and demonstrate that there is no more need to abandon the concept that growth is driven by a finite turgor pressure acting at the tip, in favour of other hypotheses [58], [59] than there is to invoke unsubstantiated faults with the plasmolytic method of determining ΔΠ [14], [56].

Changes in other independent variables directly related to turgor also cause changes in the linear extension rate, *dL/dt*. One such variable is the osmotic pressure; using the model, if the external osmolarity *C_o_* is raised the turgor pressure shows no change but the growth rate is lowered (Figure 5.G-I) therefore the model predicts that there should be no simple correlation either between pressures Δ*P* and ΔΠ or between Δ*P* and linear growth rate *dL/dt* as demonstrated experimentally by pressure-probe studies [14].

### The Expanding Tip and Changes in External [Ca^2+^] or Π

Both natural and experimental changes can directly affect calcium concentration and Π_o_ and thus two key parameters of tip growth: wall hardening and water ingress. The effects of such changes are therefore important tests of any model that seeks to explain the mechanics of tip growth.

#### Calcium ions: Simulations

In these the behaviour of other variables was monitored to give insight into the internal interactions (Figure 3). We set the wall thickness for bursting close to zero (0.2 nm) and simulated the binding of calcium ions in the medium by modulating the pectin hardening factor *α* (Eq.5). *α* is linearly proportional to the calcium concentration and also proportional to the square root of *K_c_*, the binding constant for calcium in the pectin tip gel, and the diffusivity *D*

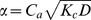
(10)(see Supporting Information S1). Experimentally *α* can be manipulated by adding or removing calcium or by adding exogenous methylesterase, which increases the production rate of carboxyl groups in the steady-state and thus *K_c_*. It can be seen that setting *α* to zero produces rapid bursting (Figure 3A-C). When *α* is made 20 times higher the model predicts that the wall thickens, causing growth to slow and then cease (Figure 3D-F). However, when the calcium ion concentration (i.e. *α*) is raised or lowered by a moderate amount the model shows that the wall thickness undergoes a perturbation but stabilizes at its former value again – unexpectedly the steady state thickness is predicted not to change over a wide range of *α* (Figure 3G-I).

The wall thickness reached after a manipulation is dependent upon a decrease in the rate of supply, *dVwall*/*dt*, when it is under feedback control. This is controlled by a time-constant in the model (see Model implementation, below) which represents the response time of the cell signalling chain between sensor and vesicle deposition. When growth becomes zero the thickening is predicted to stop, provided that the sensor inhibits cell wall deposition. Furthermore, the thickness attained would be expected to vary between tubes since there is likely to be a range of values for true parameters such as tube radius *r*, the permeability *P_os_*, and the hardening *α*, which are outside control by feedback within the model.

If there is no sensor/regulator (Eq.7) and pectin supply *dV_wall_*/*dt* has a constant value that results in the initial observed wall thickness of 0.2–0.3 µm in steady-state growth, then both thickness and growth rate become unstable and vary according to every calcium concentration change in the medium, behaviour which is not consistent with observations and would be biologically undesirable. Feedback as given by Eq.7 therefore stabilizes the wall at the tip.

#### Calcium ions: Experiments

When free calcium ions were removed from the medium by adding the chelating agent BAPTA (500 µM, Methods section) growing *Lilium* tube tips immediately commenced bursting with 70% burst by 5 mins (Figure 4). Because pollen tubes were growing on and into solidified medium, additions to the external medium are not instantly perceived by all tubes; the time course of Fig. 4 is consistent with the estimated time for the external calcium to fall at varying (shallow) depths in the medium. At the other extreme, high calcium slows growth, often to zero [13], [79], [80], and results in a thickened wall at the tip [79], [80]. When we germinated pollen in 2 mM calcium medium we observed thick walls at the tip by TEM, many being grossly thickened. A similar effect has been observed after adding exogenous pectin methylesterase to growing *Nicotiana* tubes, when the growth slowed and the tip thickened in a dose-dependent manner [81]. This is likely to be due to the increased production rate of ionized carboxyl groups in the pectin which is no longer under the control of the exocytotic cycle.

Between these extremes of calcium concentration that produce bursting or thickening lies a range in which most pollen tubes have walls of stable thickness [29] i.e. they show little variation in thickness *L_wall_* between tubes. We may thus assume that wall thickness, in conformity with the model, is stable with respect to moderate variations in calcium ion concentration in the medium. We changed [calcium] around growing *Lilium* tubes in the range 0.1–1.0 mM and observed no significant changes in their appearance by light microscopy.

#### Osmotic pressure: Simulations

When ΔΠ is increased by lowering the external osmotic concentration *C_o_*, the model predicts an increase in growth rate but the turgor ΔP and the wall thickness *L_wall_* do not change when a sensor (Eq.7) is present (Figure 5.A-C). Large decreases in ΔΠ cause bursting of the tip (Figure 5.D-F). Decreasing ΔΠ by raising *C_o_* leads to a slowing of growth (Figure 5.G-I) but no change in wall thickness unless *C_o_* is raised enough to lead to the onset of plasmolysis, in which case growth ceases. When the sensor is absent the wall thickness is not stabilized but varies with the external osmotic pressure.

#### Osmotic pressure: Experiments

The behaviour with a sensor is very similar to the experimental findings in this study and to other observations. Dilution of the medium causes bursting with the fraction bursting increasing with the extent of dilution. Rises in external osmolarity *C_o_*, which decrease ΔΠ, rapidly inhibit growth but the wall thickness shows no significant change between different osmolarities of growth media: in medium of 0.3 OsM, thickness *L*_wall_ = 0.25±0.07 µm (SD); in 0.6 OsM medium, *L*_wall_ = 0.20±0.04 µm (SD).

This indicates an essential difference between expansion growth and tip growth: the former rarely if ever shows bursting when the medium is diluted; tip growth, where water is far from equilibrium, can show bursting at raised ΔΠ due to thinning and structural failure of the tip wall. Also, both simulation and experiments show that with moderate increases in ΔΠ the tip is stable, but with high steps the increase in growth rate thins the tip wall to bursting point. The model predicts that under severe hypotonic shock the tip wall thickness begins to oscillate rapidly before bursting, which is in agreement with observations on the thinning of thin films [82], [83]). (This aspect of tip stability, together with others that lead to bursting, is discussed below in Model implementation).

## Discussion

### Control of the Wall Thickness

For clarity, we define the cell wall sections that are being considered: we will here refer to shank, tip (the curved region), and apex (the flatter portion around the tube axis). The model describes steady-state growth and does not *per se* generate oscillations. In oscillatory growth localised variations in tip thickness at the limit of light microscopical resolution have been measured at the apex [11]. However, it can be seen that these thickness changes die out on moving from apex to tip and may well represent fluctuations in pectin supply during oscillatory growth as suggested and as described by Eq.7. The model presented here is one in which the apical disc is well-mixed and so would not reveal fluctuations of this sort; the wall thickness *L_wall_* is actually the pectin volume leaving the edges of the apical disc divided by 2π*r*, where *r* is the tube radius. These fluctuations are not passed to the tip or shank and the wall thickness of both of these is remarkably constant in any cell as seen by electron microscopy, (TEM) (Figure 2); we define the stability of the wall as that process which guarantees constant wall thickness of the cell.

The important point is that, in this model, growth fluctuations must involve a periodic supply of pectin to the apical disc and any fluctuations in thickness seen there, and which cycle about a constant mean value [11], are due to the feedback interactions described in the model equations. These ensure that the apical disc ultimately feeds volume to the tip-shank wall at a constant rate per unit increase in cell volume, with or without growth oscillations. As will be shown below, growth oscillations are unlikely to be due to periodic deposition of pectin *alone* (see ***Growth oscillations***). There must be what has been called a ‘pacemaker’ for the oscillations [28] with pectin supply being an intermediate link.

### Shank Impermeability and Wall Structure

The principal component of the pollen wall along most of its length is callose, a β-1,3 glucan polymer [84] and this is found in the inner layer of the wall adjacent to the membrane but is not present at the tip [63], [85], [86] and thus is a candidate for the solute-impermeable layer of the tube wall. Callose is synthesized by callose synthase (CalS) and it appears that, in *Arabidopsis*, there are many CalS genes but a unique form (CalS5) is present in the pollen tube [87] and one also in *Nicotiana* pollen [88]; in this latter system it appears that the callose has no 1,6 branching [88], [89] unlike most callose which is a 1,3–1,6- β glucan. Callose layers have been considered to confer solute impermeability [90]–[92] and water impermeability [93] but the precise physiological properties of callose are unexplored.

### Shank Impermeability and Currents Through the Wall

If the shank is impermeable to water and water-soluble molecules such as Neutral Red and Calcein, its ion permeability should be very low, which appears to contradict reports of ion currents crossing the wall along its full length. Potential measurements with vibrating electrodes in the field around growing *Lilium* tubes have been extrapolated to ion currents flowing through the shank wall [74], [94], [95]. However, this extrapolation is only possible if the current lines are normal to the surface and this is probably untrue except for the extremities of tip and grain pore; the experimental measurements, however, are fully consistent with an impermeable shank as explained in the Supporting Information S1 and Figure S3.

### Tip Impermeablization is Expected to Interact with Ion Fluxes

As the tip extends at a rate *dL/dt*, a chemical change in the wall resulting in solute and water impermeability must also proceed towards the tip at the same overall rate for the osmotic zone to remain at a constant average length, *L_perm_* (whose area is equal to *A_mem_* of Eqs.1 and 3). In the simplest terms
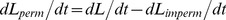
(11)where *dL_perm_/dt* and *dL_imperm/_dt* are the linear growth rates of the permeable and impermeable tube sections (Figure 7).The quantities in Eq.11 may be regarded as average rates over time but they may not be in phase in which case *L* and *L_perm_* will oscillate.

There are events occurring in the tip zone [74], [95] which strongly suggest that ‘H^+^ dynamics may underlie basic mechanisms of polarity and spatial regulation in growing pollen tubes’ [74]. An influx of proton ions at the tip creates apical acidity but this falls after a short distance distally and this fall is created by H-effluxes associated with a novel H-ATPase (NtAHA) found in *Nicotiana* pollen tubes but which is not expressed in the acidic tip [74]. However, H-pump expression must advance at an average rate equal to that of the tip itself.

The expression of the pump and the rise of alkaline conditions is likely to be associated with callose insertion into the wall because it occurs at the tip-shank junction; wall callose is confined to the shank and absent from the tip [63], [85], [86]. A special callose synthase of *Nicotiana* has a pH optimum of 7.5 falling off steeply at acid pHs [88] which would tend to inhibit callose incorporation at the acid tip. During tip growth the proton concentration in the cytoplasm is lowered - the rates of the two are apparently negatively coupled [95] - which allows the alkaline zone to advance by expressing the proton extrusion pump further towards the tip, and would favour callose synthesis in the adjacent wall, extending the impermeabilization process. We suggest that during these coupled events it is possible that time lags between rates of growth *dL/dt* and impermeabilization *dL_imperm_/dt* result in an oscillation of *L_perm_*. The possible significance of changes in *L_perm_* are examined below.

In the study of currents flowing into the apical region of *Lilium* [94] it was found that the steady current began to pulsate: “An endless train of spontaneous, monophasic pulses ride on top of the steady current … the sink for pulse current is more concentrated towards the growing tip than is that for the steady current.”. These pulsations have the same frequency as the oscillations in growth rate, about 1.1–1.3 min^−1^. The important observation here is that the steady current, which does not rise as would be expected if the tip membrane area were steadily expanding, increases and then falls back to the same value. This is consistent with a constant membrane area for ion entry that expands and contacts regularly with the advance of the tip.

### Other Possible Sensors

**Figure 10:**
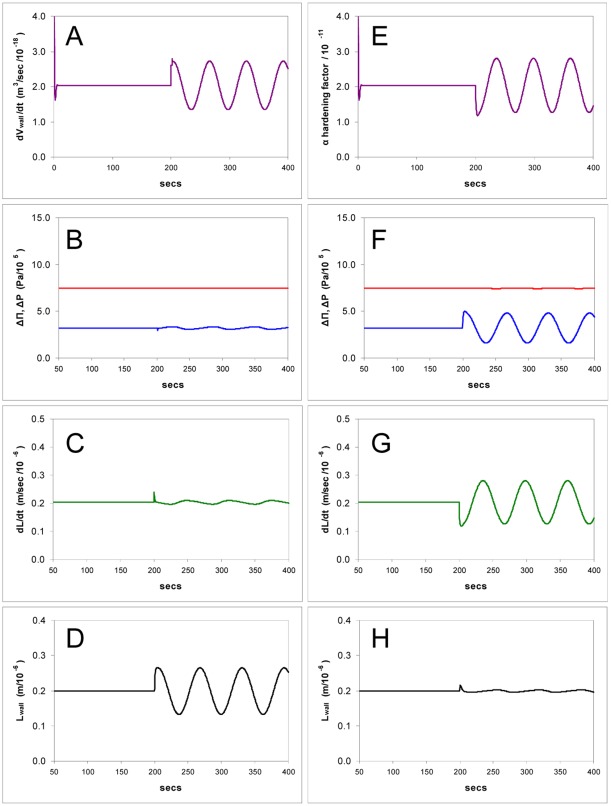
Simulation of the model showing computed time courses of the growth of a tube when the pectin extrusion rate (*dV_wall_*/*dt*) is varied sinusoidally. **A-D**: Small oscillations are observed in turgor pressure (B) and growth rate (C) with larger ones in wall thickness (D). **E-H**: As in A-D except that the hardening factor *α* is now varied sinusoidally (E) which leads to substantial oscillations in turgor pressure (F) and growth rate (G) with small ones in wall thickness (H).

**Figure 11:**
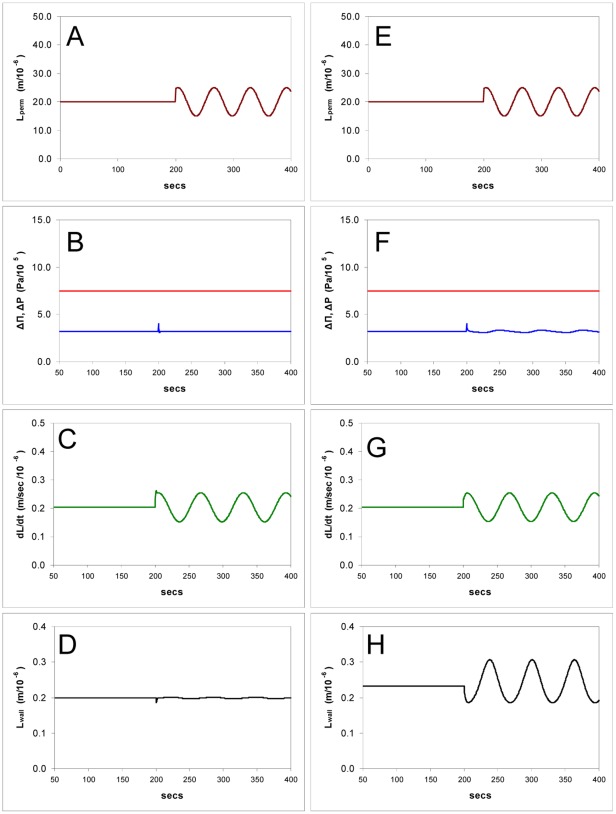
Simulation of the model showing computed time courses of the growth of a tube when the length of the osmotically active zone (permeable region of the tube *L_perm_*) is varied sinusoidally in a tube having a sensor. **A-D**: Growth rate oscillates (C) but the turgor pressure (B) and wall thickness (D) are stable. **E-H**: without the sensor, showing oscillations in turgor pressure (F), growth (G), and wall thickness (H).

In Table 1. we have collected the results of changes in certain variables during steady-state growth, with and without a sensor whose input is the water potential difference, (ΔΠ - Δ*P*). It can be seen in the simulations shown (Figs.3, 5, 10 and 11) that turgor pressure is regulated by the physico-chemical properties of the model equations without a sensor, but the wall thickness is not. The problem of wall stability, i.e. controlling the thickness of a liquid film that is expanding fast, cannot be solved by leaving the wall extrusion process to proceed independently, at a constant rate, in the face of changes in calcium concentration or ΔΠ.

**Table 1:** The effect of perturbations and sensor models on the key parameters of growth compared with experimental observations.

Perturbation	Sensor feedback	Growth rate changes	Tip wall thickness changes	Turgor pressure changes	Compatible with observation
Π*_o_*	no	yes	yes	no	no
*α* [Ca]	no	yes	yes	yes	no
*L_perm_*	no	yes	yes	yes	no^4^
Π*_o_*	yes	yes	no^1^	no	yes
*α* [Ca]	yes	yes	no^2^	yes^3^	yes
*L_perm_*	yes	yes	no	no	yes^4^

Growth rate (*dL*/*dt*), tip wall thickness (*L_wall_*), and turgor pressure (*ΔP*) were computed from the model in response to different perturbations and compared to values measured in this study or observations in the literature. External osmotic pressure Π*_o_* and hardening *α* can be experimentally manipulated under physiological conditions but zone length *L_perm_* cannot. The magnitude of simulated perturbations, whether step changes or sinusoidal, are at least 50% of the steady-state values. Only the model with an osmosensor of (ΔΠ–Δ*P*) and the output as pectin exocytosis *dV_wall_/dt* fits the experimental observations.

1 Wall thickness is constant until very low values of Π*_o_* (hypotonic challenge) whereupon it becomes unstable and bursting occurs.

2 Wall thickness is constant until [Ca] approaches zero when it becomes unstable and bursting occurs; at high concentration ([Ca] = 2 mM) the growth slows to zero during which time the wall thickens substantially.

3 Turgor pressure change with Ca changes have not been measured in pollen to our knowledge; however, increased wall plasticity induced in fungal hyphae do lead to a fall in turgor [56] as would be expected.

4 If periodic changes in this parameter are occurring during growth.

A sensor can detect a change in an intensive variable, such as ΔΠ or Δ*P* (which might be detected by osmosensors or mechano-sensors respectively), but not the main rate parameters: solute accumulation rate *dm/dt*, hardening rate *α*, or pectin extrusion rate *dV_wall_*/*dt*, which have to be independently set for the model to run. Conversely, a sensor cannot detect a parameter that it sets itself, nor can it directly control intensive variables such as ΔΠ or Δ*P* which are determined by other parameters as described by Eqs.1–6. The variables such as wall thickness *L_wall_*, or growth rate *dL*/*dt*, are also controlled by the values of other parameters in the overall model equations. Therefore we considered three possible sensor inputs ((ΔΠ - Δ*P*), ΔΠ, or Δ*P*, and three sensor outputs of pectin extrusion, solute accumulation and hardening (*dV_wall_*/*dt, dm/dt*, and *α*) and analysed the nine resulting possibilities for coupling.

Coupling of (ΔΠ - Δ*P*) to deposition *dV_wall_*/*dt* has been a main consideration of this paper, so it remains to consider the behaviour of the other eight possibilities. It can be said immediately, as a result of many simulations that were run, that the couplings (ΔΠ - Δ*P*) to *dm/dt*, ΔΠ to *dm/dt*, and Δ*P* to *dV_wall_*/*dt, dm*/*dt* or *α*, could not be made to give any steady-state growth pattern and were therefore eliminated. Model versions using the remaining three couplings, (ΔΠ - Δ*P*) to *α*, ΔΠ to *dV_wall_*/*dt*, and ΔΠ to *α*, (coupled in a form of Eq.7) were able to simulate steady state growth. They were next tested with respect to perturbation of the two experimentally accessible parameters: changes in hardening *α* (by changes in external Ca^+^ concentration) or in ΔΠ (by changes in solute concentration *C_o_*). All three alternative sensor versions caused immediate changes in turgor and wall thickness after perturbing either *α* or ΔΠ. We conclude that turgor pressure and wall thickness are only stabilized by a sensor that detects (ΔΠ - Δ*P*) and controls *dV_wall_*/*dt*. We therefore suggest that pollen and other tip growing cells possess sensors present in the cell membrane that detect the water potential gradient and whose signals regulate cell wall deposition rates.

### Growth Oscillations

Oscillations of growth rate, ion currents and pH levels have been repeatedly observed in growing pollen tube tips [2], [50], [74]. However, these oscillations do not always occur in actively growing tubes, even within the range of species and tube lengths where they are commonly seen; thus oscillations are not needed for growth, and may be purely epiphenomenal. There is general agreement that they are manifest in tubes with greater length. Nevertheless, they are a phenomenon that an integrated model of tip growth should be able to accommodate. Simulations show that when any of several of the independent variables of the model presented here is perturbed about a mean value with the approximate frequency of the observed growth oscillations (∼ 1 min^−1^) oscillations in growth rate are observed. Dependent variables such as turgor pressure and cell wall thickness may be seen in simulations to oscillate as well with the same frequency as the driving variable. Such simulations can therefore be used as a valuable tool to investigate the connections between variables.

Measurements along the wall of growing tubes fail to show any consistent pattern of fluctuation in thickness along the wall length. In *Lilium* oscillations have a frequency of about 1.2 min^−1^ and growth rates vary but are about 12 µm/min. If there were oscillations in thickness accompanying growth, there should be bands of repeating wall thickness seen with a length of ∼10 µm. but this is not apparent. We conclude that during oscillatory growth, when the growth rate commonly varies by ±50% and the pectin delivery rate must change by about 100%, the wall thickness is stable. In this context, the composition of the wall from tip to early shank may differ, and show periodicity of different components, sometimes in bands [96]–[99], but the origin of these are obscure and they are not observations of linear variations in wall thickness.

Several mechanisms have been suggested as possible initiators of growth oscillation: changes of turgor pressure [25], [75], [100] or osmotic pressure [101], pectin extrusion rate [11], and wall plasticity [102]. Turgor pressure changes are not observed [14] even in tubes which have bifurcated; in these, the two tips can show different oscillation rates whilst the pressure is constant throughout the cell [21], [103]. Osmotic pressure variations would have to be translated into pressure oscillations to effect growth but these could not be localized to the tip due to the near-incompressibility of water [21], [104]. In all simulations involving changing osmotic pressure the turgor pressure also shows changes which are not observed *in vivo* and so we shall not consider these further.

Pectin supply to the tip *dV_wall_*/*dt* was varied sinusoidally in the simulation of Figure 10A-D which shows fluctuating pressure and wall thickness - in contrast with observations. The pectin supply rate affects the viscosity inversely (Eq.5) and the two can only be uncoupled by changing the chemistry of hardening which may conceivably fluctuate independently of the rate of pectin supply. Therefore in the simulation of Figure 10.E-H only the hardening rate *α* was varied sinusoidally, which also affects the plasticity (viscosity) by Eq.5, and now the pressure and growth rate oscillate in phase - again contradicting measurements of these parameters. While oscillations in pectin supply may be an intermediate in the chain it remains to be shown what causes such oscillations in the first place, and it must follow that pressure and wall stabilisation must be operating as part of the mechanism.

### The Possibility of a Pacemaker

In Figure 11.A-D we show the results of simulating an oscillation in the osmotic zone length *L_perm_* which leads to proportional changes in growth *Ldt*. The growth rate changes by a factor of about 2 as reported in several studies [8], [13], [75], [100] but no changes in turgor are seen. The amount of pectin entering the wall is approximately doubling during each cycle but the wall leaving the apex does not thicken. In Figure 11.E-F we show the effects of oscillating *L_perm_* without a sensor; in this case the rate of pectin extrusion does not change, and both the turgor pressure and the wall now show large oscillations. When the effect of oscillating the osmotic permeability *P_os_* of the osmotic zone was simulated, all observable variables showed very substantial fluctuations, with or without a sensor (not shown).

These model simulations show that variation in the zone length *L_perm_*, together with an sensor controlling the pectin exocytotic rate, can drive oscillations in growth rate without significant oscillations of pressure or wall thickness. As *L_perm_* is under dynamic control, and may oscillate, we suggest that it is a prime candidate for the driver of oscillations in growth rate. If the feedback controlling *L_perm_* does not oscillate under certain conditions (such as slow growth rates) Eq.11 would still apply irrespective of the precise mechanism or values of rate constants that would be involved, because of its very general nature.

In addition, the ionic currents entering the tube at the tip must enter over the area of the osmotic zone and the oscillation of these may be considered to be dependent on the magnitude of *L_perm_* and therefore driven by changes in its membrane area rather than being the initiators of the growth rate themselves. In this context it should be noted that in a recent ionic pollen model [50] which divides up the tube membrane into two sections, tip and shank, each possessing different ion pumps and transporters which drive current through the cell, the authors suggest that growth oscillations might be caused by variation in the area ratio of the two sections. This would be similar to the present suggestion where *L_perm_* alters the tip area but the rest of the cell permeability remains unaltered.

## Materials and Methods

### Pollen Experiments

Flowering stems of Lilium longiflorum were obtained from florists and kept in water at room temperature. Pollen was collected from anthers 2 days after dehiscing. Pollen was used fresh or stored at −20°C after 2 hours drying at room temperature. Stored pollen was re-hydrated in a humidified atmosphere in Petri dishes lined with wet filter paper at room temperature for 1 h before use.

#### Growth

(1) Pollen tubes were grown in the germination medium (see below) in the following ways. Medium solidified with 1% agarose on cavity slides for growth measurements and for following the effects of 500 µM BAPTA (1,2-bis(o-aminophenoxy)ethane-N,N,N′,N′-tetraacetic acid). (2) For experiments to measure osmotic permeabilities the flow chamber was used. Pollen was then grown in fluid medium on cover slips coated with 400 µg/ml poly-D-Lysine (M.W. 70–150 K). (3) For confocal microscopy pollen was grown in plastic Petri dishes of 35 mm diameter with a cover slip base. The glass was coated with Poly-D- Lysine at 400 ug/ml or a thin layer of 1% agarose. The range of growth rates was the same in fluid and solid media.

#### Media

Basic ionic medium: KCl 1 mM, CaCl_2_ 0.1 mM, H_3_BO_3_ 1.6 mM, MES/Tris 5 mM, pH 5.5–5.6. Basic Growth medium: basic ionic medium plus sucrose 300 mM. Osmotic pressure was varied by changing the sucrose concentration.

#### Flow chamber

The flow chamber and accessories (suitable for imaging using a confocal microscope) was purchased from Warner Instruments [Harvard]. Large 250 um gaskets were used as spacers. Flow was gravimetric from 20 ml syringes. Tubing, a chamber perfusion manifold and valves were used to regulate the flow rate at 0.4 ml/min.

### Microscopy

Pollen grown on solid medium in cavity slides or on coverslips for the flow chamber were used to follow and film growth and the effects of varying conditions on growing tubes using a Zeiss light microscope fitted with a Leica digital camera connected to a computer and LAS software was used for analysis. To film Calcein dye entry and the effect of HgCl_2_ on pollen tubes Leica SP2 confocal microscope was used as described below.

#### Confocal microscopy

Pollen was grown in plastic Petri dishes of 35 mm diameter with a No.0 cover slip fixed into the base with Sylguard elastomeric cement (Dow Corning). The glass was coated with Poly-D- Lysine (Sigma) at 400 ug/ml and pollen was put into 100 ul standard growth medium laid on the cover slip or on a thin layer of 1% agarose in growth medium. Pollen tubes were imaged using a Leica SP2 confocal microscope with a×20 working distance (3 mm), water immersion, objective lens with an NA of 0.5. To study dye entry into the tube Calcein (Invitrogen Molecular Probes) at 2 µM was added and time lapse series were taken both in transmitted light and to follow fluorescence. Calcein was excited at 488 nm and emitted light was captured between 490 nm and 550 nm.

#### Electron microscopy

Lily pollen tubes were grown in the media listed above either in fluid or solidified with agarose. Fixation : pollen tubes were fixed in 4% glutaraldehyde in 0.1 M Pipes buffer for 1–2 hrs at room temperature. Rinsed in 0.1 M Pipes buffer-3×5 minutes. Post-fixed in 1% Osmium ferricyanide for 1 hr. Rinsed in 0.1 M Pipes buffer-3×5 minutes. Bulk stained with uranyl acetate in maleate buffer 1 hr. Rinsed in water. Dehydration in ethanol 70% 3×5 minutes, 95% 3×5 minutes, 100% 3×5 minutes. Acetonitrile 2×5 minutes. 50% acetonitrile/50% Quetol 651 resin 1 hr. 25% acetinitrile/75% Quetol 651 resin left overnight. 100% Quetol 651 resin – no BDMA -1 day. 100% Quetol 651 resin + BDMA for 2 days. Embedded and cured at 60°C for 24 hrs. Sectioning and staining : Sections were cut at 70 nm on a Leica Ultramicrotome with a Diatome diamond knife, collected on 100 mesh copper grids or Formvar-coated copper slot grids, counterstained with uranyl acetate and lead citrate and viewed in a Phillips CM100 TEM.

### Model Implementation

The model was implemented using *Mathematica 4.0*. on desktop computers with a set of parameters and independent variables closely matching those known for growing *Lilium* pollen tubes. Unknown parameters were assigned magnitudes which resulted in dependent variable values close to those observed in normal growth, taking care to make the assignments reasonable (biologically and physically) and all the resulting variable values acceptable and consistent. This comprised the standard parameter value set of the model. After starting iterations from initial conditions (a stationary tube of length 500 µm loaded with solute at water equilibrium and a given solute accumulation rate *dm*/*dt*) a steady-state was reached which could then be perturbed by a change in a parameter value or by a relation between particular variables, according to the objective of the simulation. The model equations were advanced iteratively by time steps of 0.4 secs. Smoothing functions (exponentials with suitable time-constants) were used to control (i) abrupt perturbations in parameters, (ii) sensor feedback and (iii) the rate of expansion of the tip area *dA_tip_*/*dt* by the pressure which otherwise could cause artifactual fluctuations in growth rate and pressure at the frequency of the iterations used in forward step time. The smoothing half-times used were between 0.2–0.5 of the computational time step.

Certain treatments regularly cause bursting of pollen tubes at the tip and these were examined as simulations. In all cases bursting occurs when the tip film thickness becomes very small and begins to oscillate, approaching the zero-line. This was unavoidable in model computation using finite time iterations and probably occurs physically in all films [82], [83]; the bursting threshold was set as 0.2 nm corresponding to a sub-molecular width for membrane lipids.

## Supporting Information

Figure S1 **Entry of calcium and pectin into an expanding section of tip wall.** The primary wall is bounded by the tip membrane at left (*x* = *L_wall_*) and the bath at (*x* = 0). Pectin is extruded into this wall at a rate *dV_wall_*/*dt* and calcium ions diffuse in from the bathing medium at concentration *C_o_*. The curve shows the concentration gradient of free calcium within the tip wall.
(TIF)

Figure S2 **Plots of** ***α*** **as a function of wall thickness at different carboxyl densities and a calcium concentration in the external medium of 200 µM.** Curves were calculated using Eq.3 of section1. The wall thickness measured by TEM is close to 0.2 µm.
(TIF)

Figure S3 **Illustration of current lines around a pollen tube dipole with an impermeable shank and permeable grain and tip regions.** The gradients are shown at two points, with current components perpendicular to the pollen tube rising and falling along the axis. The current at the shank surface is zero.
(TIF)

Video S1 **Entry of Calcein AM into four Lilium tubes: sequence accelerated by 20 times.** False colour spectrum : red (low) to blue (high). Calcein appears initially in the tips; as the local concentration increases it is carried down the tubes by streaming.
(AVI)
