# Supplementary figures and images for: An Osmotic Model of the Growing Pollen Tube
Source: PLoS One. 2012 May 16;7(5):e36585. doi: 10.1371/journal.pone.0036585 (PMC3353927; doi:10.1371/journal.pone.0036585)

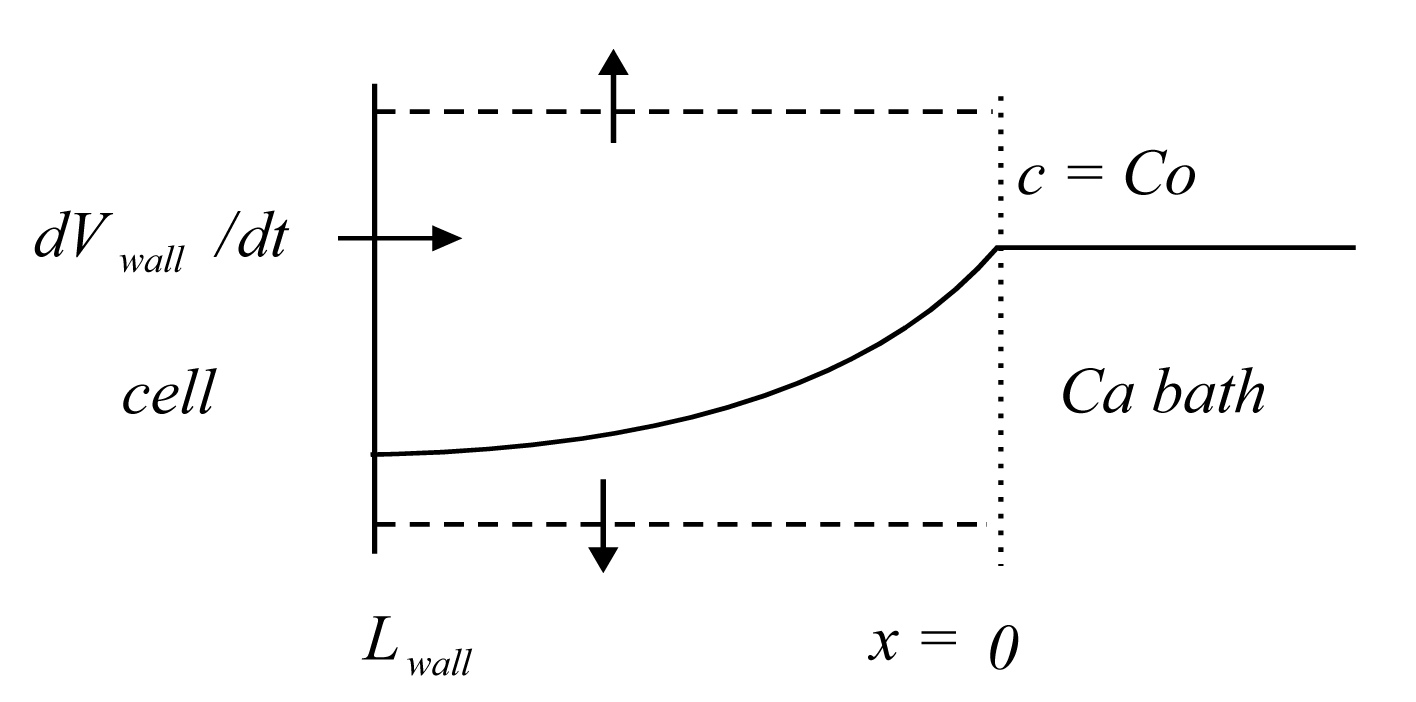

Supplement: Figure S1 — Entry of calcium and pectin into an expanding section of tip wall. The primary wall is bounded by the tip membrane at left (x = Lwall) and the bath at (x = 0). Pectin is extruded into this wall at a rate dVwall/dt and calcium ions diffuse in from the bathing medium at concentration Co. The curve shows the concentration gradient of free calcium within the tip wall. (TIF) [file pone.0036585.s001.tif]

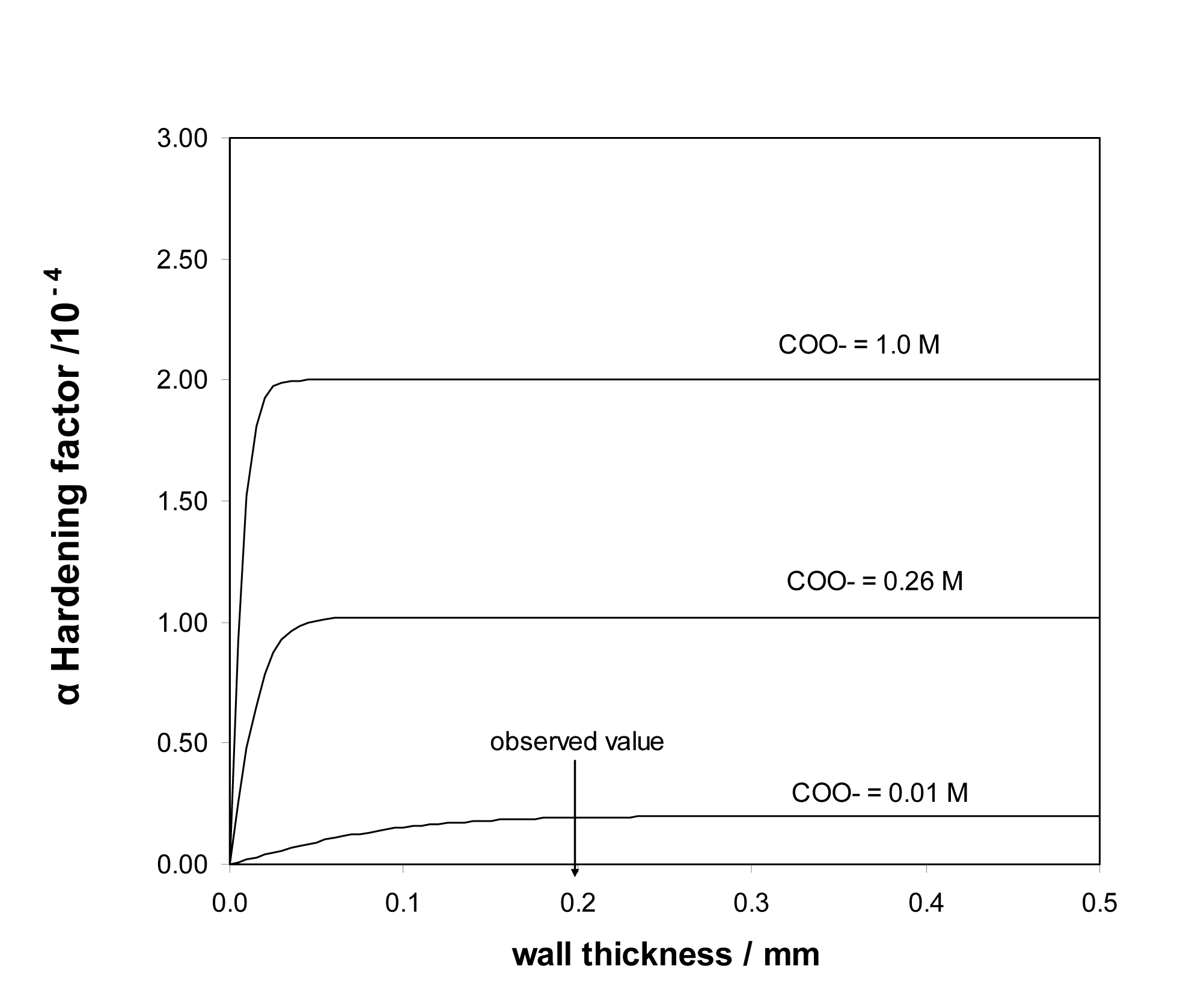

Supplement: Figure S2 — Plots of α as a function of wall thickness at different carboxyl densities and a calcium concentration in the external medium of 200 µM. Curves were calculated using Eq.3 of section1. The wall thickness measured by TEM is close to 0.2 µm. (TIF) [file pone.0036585.s002.tif]

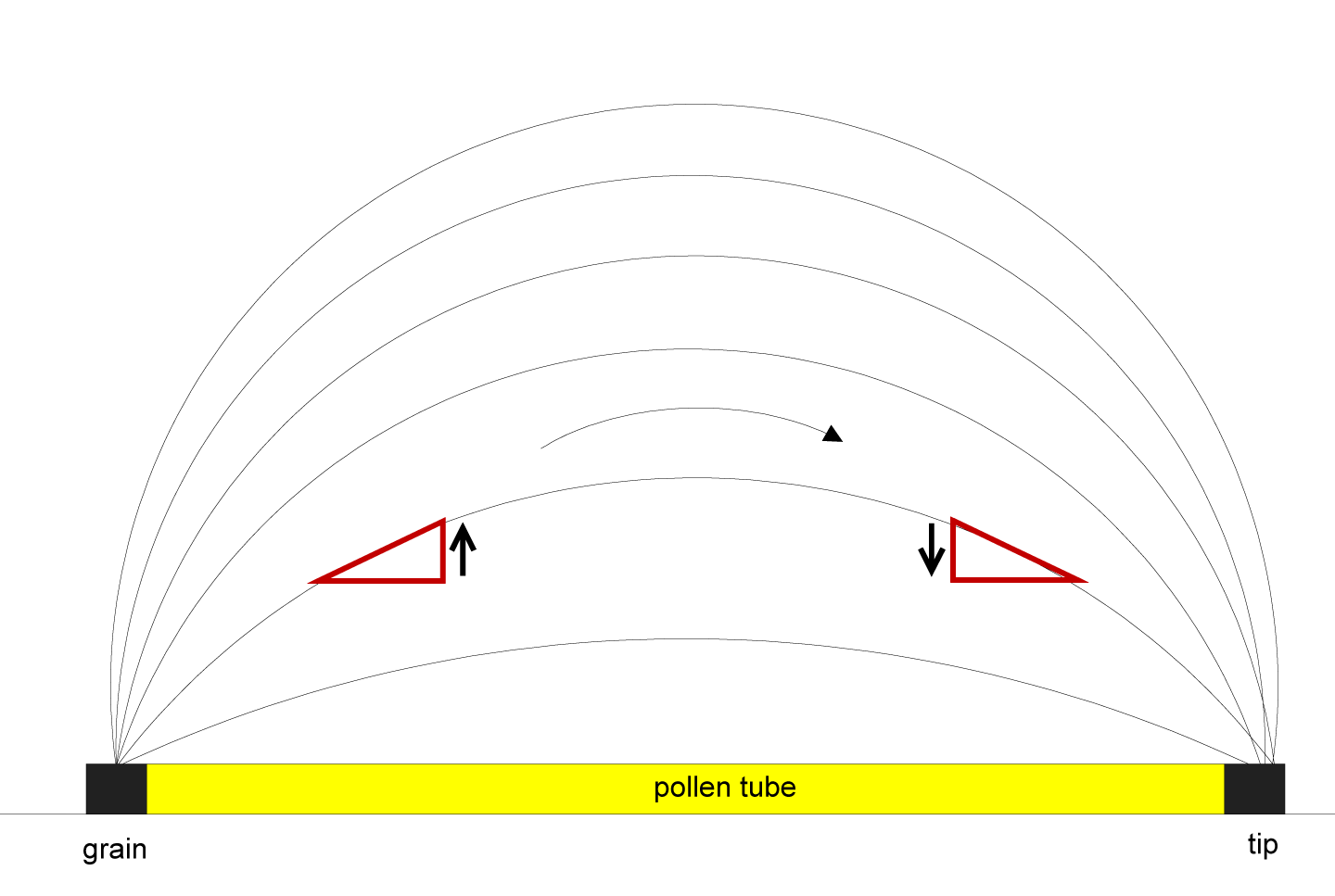

Supplement: Figure S3 — Illustration of current lines around a pollen tube dipole with an impermeable shank and permeable grain and tip regions. The gradients are shown at two points, with current components perpendicular to the pollen tube rising and falling along the axis. The current at the shank surface is zero. (TIF) [file pone.0036585.s003.tif]
